# Synthesis, Characterization, and Biological Evaluation of Nanostructured Hydroxyapatite with Different Dimensions

**DOI:** 10.3390/nano7020038

**Published:** 2017-02-15

**Authors:** Zhen Geng, Qin Yuan, Xianglong Zhuo, Zhaoyang Li, Zhenduo Cui, Shengli Zhu, Yanqin Liang, Yunde Liu, Huijing Bao, Xue Li, Qianyu Huo, Xianjin Yang

**Affiliations:** 1 Tianjin Key Laboratory of Composite and Functional Materials, School of Materials Science and Engineering, Tianjin University, Tianjin 300072, China; nanboshan1987@163.com (Z.G.);zdcui@tju.edu.cn (Z.C.); slzhu@tju.edu.cn (S.Z.); yqliang@tju.edu.cn (Y.L.); 2 School of Laboratory Medicine, Tianjin Medical University, Tianjin 300072, China; m13752208393@163.com (Q.Y.); yundeliu@126.com (Y.L.); kris_10713@126.com (H.B.); nicolelea@163.com (X.L.); huo.qianyu@163.com (Q.H.); 3 Department of Spinal Surgery, Liuzhou Worker’s Hospital, Liuzhou 545001, China; 4 School of Materials Science and Engineering, Tianjin University, 92 Weijin Road, Tianjin 300072, China

**Keywords:** nanostructured materials, surface properties, crystallization, absorbable implants, biocompatible materials

## Abstract

Nanosized hydroxyapatite (HA) is a promising candidate for a substitute for apatite in bone in biomedical applications. Furthermore, due to its excellent bone bioactivity, nanosized strontium-substituted HA (SrHA) has aroused intensive interest. However, the size effects of these nanoparticles on cellular bioactivity should be considered. In this study, nanosized HA and SrHA with different dimensions and crystallization were synthesized by hydrothermal methods. The phase, crystallization and chemical composition were analyzed by X-ray diffraction (XRD) and Fourier transform infrared spectroscopy (FT-IR), respectively. The morphology was observed under field emission scanning electron microscopy (FE-SEM) and transmission electron microscopy (TEM). The degradation behaviors of the samples were monitored by determining the ions release profile with inductively coupled plasma mass spectrometry (ICP-MS). The releasing behavior of Ca^2+^ and Sr^2+^ showed that the degradation rate was proportional to the specific surface area and inversely proportional to crystallization. The in vitro experiment evaluated by MG63 cells showed that SrHA nanorods with a length greater than 100 nm had the best biological performance both in cell proliferation and differentiation (* *p* < 0.05 compared with HA-1 and SrHA-1; * *p* < 0.01 compared with HA-2). In addition, HA nanoparticles with a lower aspect ratio had better bioactivity than higher ones (* *p* < 0.05). This study demonstrated that nanosized HA and SrHA with subtle differences (including dimensions, crystallization, specific surface area, and degradation rate) could affect the cellular growth and thus might have an impact on bone growth in vivo. This work provides a view of the role of nano-HAs as ideal biocompatible materials in future clinical applications.

## 1. Introduction

Due to its good biocompatibility, bioactivity, and osteoconductivity with human body constituents, hydroxyapatite (HA, Ca_10_(PO_4_)_6_(OH)_2_), which is the main inorganic constituent of human bones and teeth, has been widely used in biomedical applications, such as tissue engineering systems [1], replacements for bony and periodontal defects [2], bioactive coating on metallic osseous implants [3], and dental materials [4]. Many clinical studies have successfully proved that HA shows good performance in terms of osseous ingrowth [5,6,7]. Among the various HA structures, nanosized HA, which has a high surface activity and an ultrafine structure, similar to the mineral found in hard tissues [8], has been the most promising material for a variety of biomedical applications [9,10]. Many studies have shown that nanosized HA exhibits enhanced resorbability and higher bioactivity than micron-sized HA [11,12,13]. In addition, nanosized HA shows improved densification and sinterability due to its high surface energy [14,15]. Moreover, studies have shown that nanosized HA possesses a significant capability of improving osteogenesis-related cell proliferation and differentiation [16,17]. Therefore, nanosized HAs have aroused intensive interest, and great efforts have been made to study their synthesis, structure, and properties [11,18].

Native bone tissue is a composite composed of calcium phosphate (CaP) salts and a collagen fiber matrix and contains various cations, such as Si^4+^, Mg^2+^, and Zn^2+^ [15,19,20]. Hence, introducing extrinsic cations into the HA lattice is an effective method commonly used to improve its biological performance [21]. Strontium (Sr) is a trace element of the human body, of which 98% can be found in the skeleton. Many studies have proved that Sr not only enhances the proliferation of osteogenesis-related cells and bone matrix synthesis but also reduces the osteoclast activity [22,23,24]. Sr has also been shown to stimulate bone formation and decrease bone resorption in both animal studies and clinical trials [25,26]. For these reasons, Sr-substituted HA (SrHA) has attracted considerable interest from researchers and clinicians. However, to the best of our knowledge, the effect of morphology on the bioactivity of HA and SrHA is not yet known. Shi et al. found that HA nanoparticles with diameters of ~20 nm were better than those of ~80 nm in terms of the promotion of cell growth [27]. Another study by Mohandes et al. showed that the bioactivity of the one-dimensional HA nanostructures is greater than that of the zero-dimensional HA nanostructures [28]. Consider that the dimensions of apatite crystals in natural bone are stable, approximately ~50 × 25 × 2 nm [29]. Therefore, it is necessary to research the biological properties of nanosized HA and SrHA with subtle differences in dimensions.

## 2. Results

### 2.1. Results of Sample Characterization

**Figure 1:**
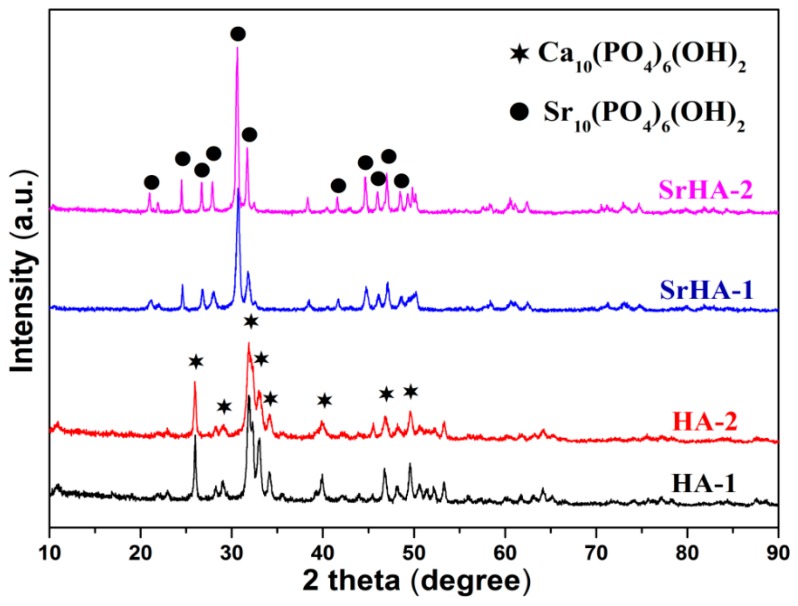
X-ray diffraction (XRD) patterns of the prepared samples. SrHAs exhibited sharper diffraction peaks than those of HAs, those indicating a higher crystallinity.

The typical XRD patterns of all synthesized samples are shown in Figure 1. The patterns indicated that the main phase for HA-1 and HA-2 was Ca_10_(PO_4_)_6_(OH)_2_ (JCPDS03-0747). Meanwhile, the main phase for SrHA-1 and SrHA-2 was Sr_10_(PO_4_)_6_(OH)_2_ (JCPDS12-0361). It was also revealed that SrHA-1 and SrHA-2 exhibited sharp diffraction peaks, especially for SrHA-2, indicating a high crystallinity. There were no significant differences between HA-1 and HA-2 with respect to the diffraction peaks. The crystallinity results calculated by Equation (1) indicated that SrHA-2 had the highest degree of crystallization (83.7%); it was 71.9% for SrHA-1. The crystallization degree of both SrHA-2 and SrHA-1 was significantly higher than that of HA-1 (55.4%, *p* < 0.01) and HA-2 (35.6%, *p* < 0.01), as shown in Table 1.

**Figure 2:**
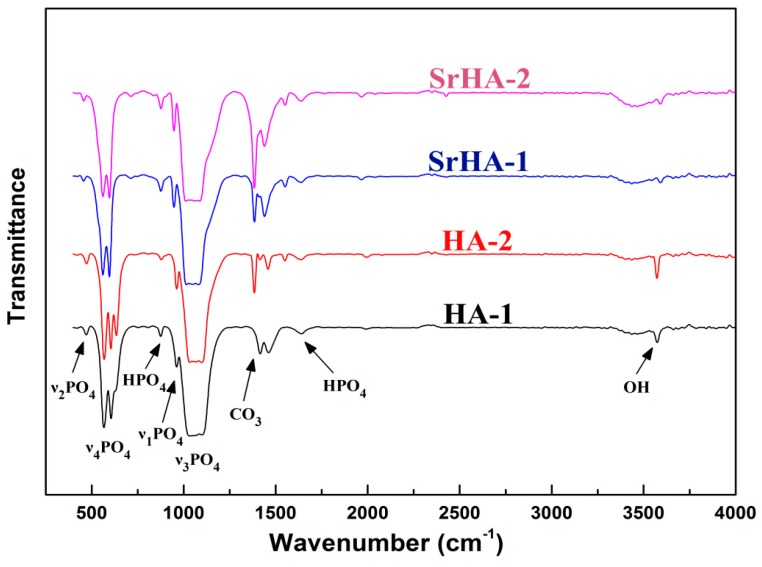
Fourier-transform infrared spectroscopy (FT-IR) spectra of the prepared powders. The main component of all samples is phosphate. SrHAs showed strengthened carbonate absorption intensity compared to HAs.

Figure 2 shows the FT-IR spectra of the prepared samples. The absorption bands at 1000–1100(υ_3_), 963(υ_1_), 577–603(υ_4_), and 472(υ_2_) detected in the spectrum, which were attributed to the phosphate (PO_4_^3−^) characteristic absorption, were present in all the spectra for the synthesized samples. The absorption band at 1640 cm^−1^ was attributed to hydrogen phosphate, HPO_4_^2−^. The absorption band at 1390 cm^−1^, which was derived from the vibration of the CO_3_^2−^ group, was owing to carbonated hydroxyapatite. The carbonated hydroxyapatite was derived from the reaction of the PO_4_^3−^ group and CO_2_ in the air. The absorption band at 3570 cm^−1^ was assigned to the characteristic OH vibrations (stretching frequency) of HA. The carbonate absorption intensity was strengthened by the incorporation of Sr (SrHA).

**Figure 3:**
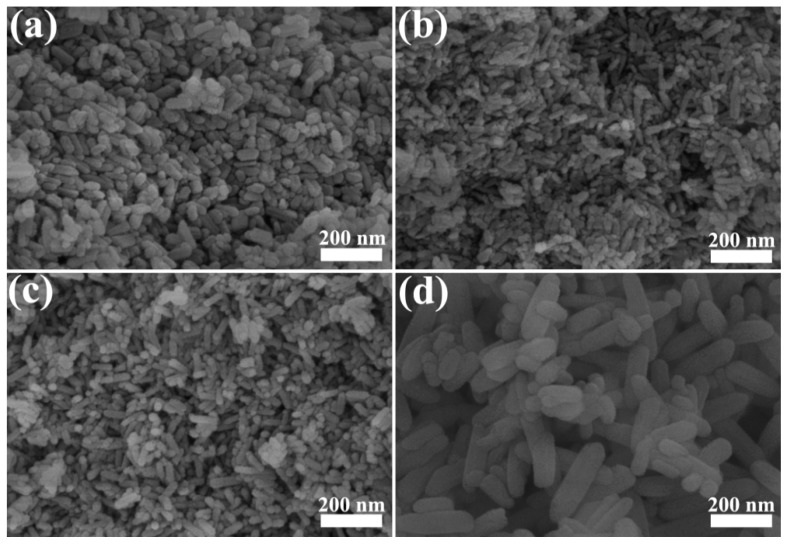
Scanning electron microscopy (SEM) images of the synthesized samples: (**a**) HA-1; (**b**) HA-2; (**c**) SrHA-1; and (**d**) SrHA-2. SrHA-2 exhibited a nanorod shape and had a larger size than the other three.

**Figure 4:**
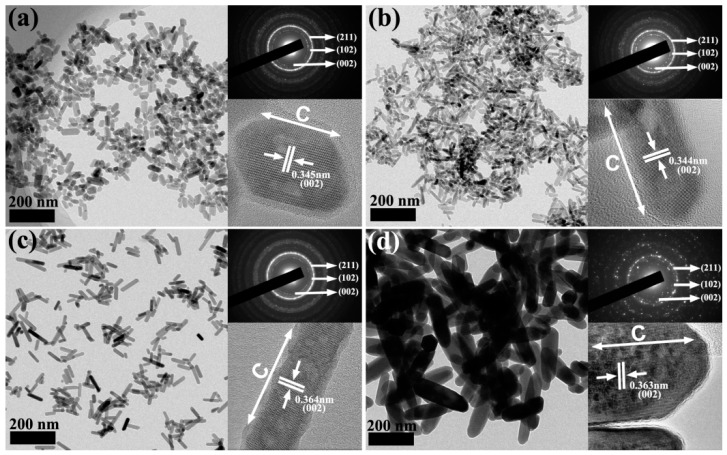
Transmission electron microscopy (TEM) and selected-area electron diffraction (SAED) patterns of the synthesized samples: (**a**) HA-1; (**b**) HA-2; (**c**) SrHA-1; and (**d**) SrHA-2.

SEM and TEM were used to study the morphology of the prepared samples, as shown in Figure 3 and Figure 4. The SEM images showed that the morphologies of HA-1, HA-2, and SrHA-1 were nanoparticles (less than 100 nm, Figure 3a–c), while SrHA-2 was a nanorod larger than the other three (Figure 3d). This was further confirmed by the TEM (Figure 4). Meanwhile, SAED and HRTEM patterns demonstrated that all samples had good crystallinity, which was in agreement with the results of XRD.

**Figure 5:**
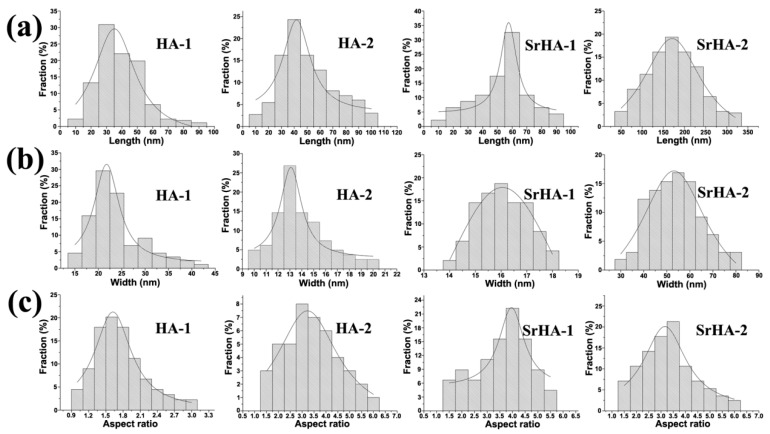
(**a**) Length distribution of HA and SrHA; (**b**) Width distribution of HA and SrHA; (**c**) Aspect ratio distribution of HA and SrHA.

For the sake of comparison, the distribution of length, width, and aspect ratio of the samples were manually measured, as shown in Figure 5. It was evident that the length of SrHA-2 (approximately 100–300 nm) was higher than that of the other three (lower than 100 nm). Meanwhile, SrHA-2 had the largest width (40–80 nm); HA-1 took the second place (15–40 nm), and HA-2 and SrHA-1 were the smallest (10–20 nm). In addition, HA-2, SrHA-1, and SrHA-2 had a similar aspect ratio (1.5–6.0), while it ranged from 1.0 to 3.0 for HA-1. These results indicated that both the length and width of SrHA-2 are significantly larger than those of the other three (*p* < 0.01). HA-2, SrHA-1, and SrHA-2 showed a similar aspect ratio, which was significantly bigger than that of HA-1 (*p* < 0.01). The BET analysis showed that the specific surface area of HA-2 was the biggest (92 ± 8 m^2^/g) and significantly higher than the other three (*p* < 0.01). Meanwhile, SrHA-2 was the smallest (38 ± 4 m^2^/g). In addition, it was 57 ± 6 and 45 ± 5 m^2^/g for HA-1 and SrHA-1, respectively, as shown in Table 1.

**Figure 6:**
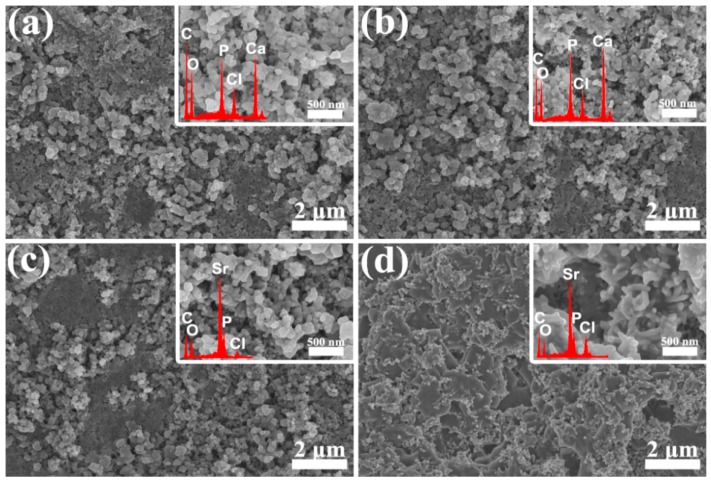
SEM morphology of synthesized coatings: (**a**) HA-1, (**b**) HA-2, (**c**) SrHA-1, and (**d**) SrHA-2. The SrHA-2 coating showed a nanorod morphology (inset), which was different from the other three (nanoparticles).

In order to investigate the effect of the samples on cellular bioactivity, Ti-based nanoparticle coatings were prepared, as shown in Figure 6. Based on SEM observations, all coatings had uniform surface structures. Meanwhile, the SrHA-2 coating showed a nanorod morphology, which was different from the other three (nanoparticles), as shown in the inset. 

### 2.2. Ion Release Property

**Figure 7:**
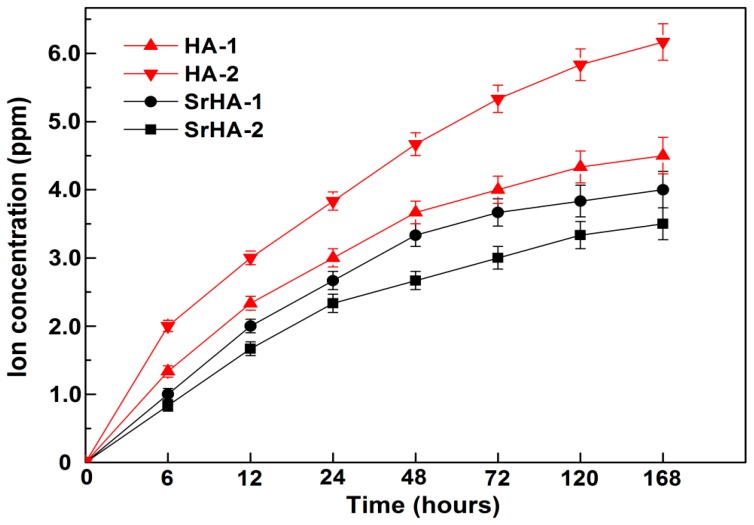
Cumulative Ca^2+^ (red line) and Sr^2+^ (black line) ion release as a function of time.

The Ca and Sr release kinetics are measured over the course of seven days, as shown in Figure 7. It is evident that the releasing rates of all samples are rapid in the early stages and slow over time. Meanwhile, the released Ca amounts of HA-1 are significantly higher than those of HA-2 during the whole process (*p* < 0.01). In addition, SrHA-2 shows a slower releasing rate than SrHA-1 (not significant).

### 2.3. Cell Adhesion, Proliferation, and Alkaline Phosphatase Activity

**Figure 8:**
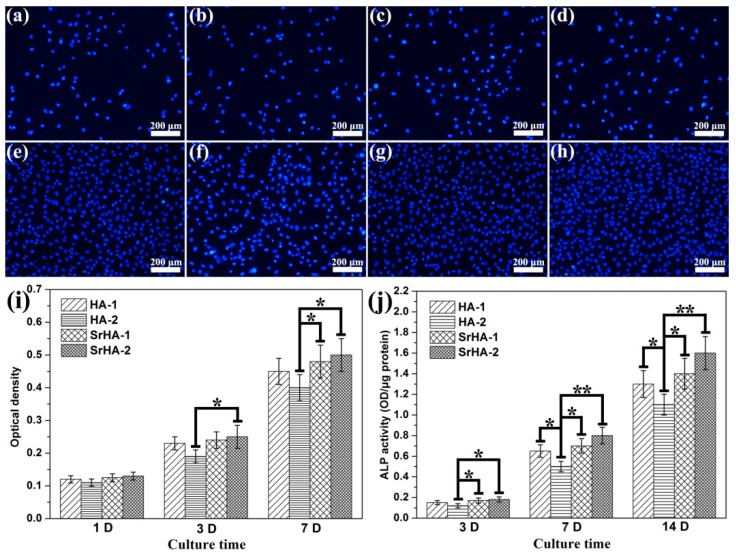
MG63 cell adhesion and distribution after one (**a**–**d**) or three (**e**–**h**) days of incubation. (**a**,**e**) HA-1, (**b**,**f**) HA-2, (**c**,**g**) SrHA-1, and (**d**,**h**) SrHA-2. (**i**) MG63 cell proliferation after one, three, and seven days of incubation measured by colorimetric MTT assay. (**j**) ALP activity of MG63 cells after three, seven, or 14 days of incubation. Statistically significant differences (* p < 0.05, ** p < 0.01).

Cell adhesion and distribution after one and three days of incubation, respectively, are shown in Figure 8a–h. There is no obvious difference between all samples after culturing for one day. However, after three days’ culture, HA-2 had the lowest cell density. This was confirmed by the MTT analysis (Figure 8i). After three or seven days’ culturing, the cell proliferation on SrHA-2 was significantly higher than that of HA-2. The results from the ALP activity test indicate that MG63 cells cultured on the HA-2 samples have a significantly lower ALP activity than that of other samples (Figure 8j). Moreover, MG63 cells cultured on the SrHA-2 samples show higher ALP activity than those on HA-1 and SrHA-1 (not significant).

### 2.4. Osteogenesis-Related Gene Expressions

**Figure 9:**
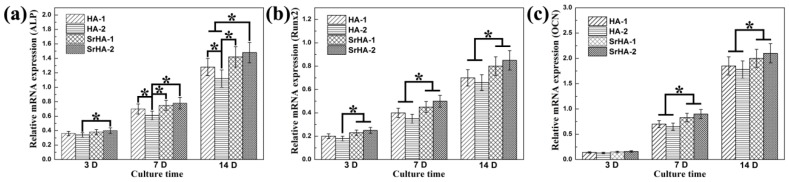
Gene expressions of MG63 cells cultured on different samples after incubation for three, seven, or 14 days: (**a**) ALP; (**b**) Runx2; (**c**) OCN. Statistically significant differences (* *p* < 0.05).

The expressions of osteogenesis-related genes, including ALP, Runx2, and OCN, in cells cultured on different samples for three, seven, and 14 days are quantified by real-time PCR and the results are shown in Figure 9a–c. The gene expressions of ALP, Runx2, and OCN on SrHA-2 are the highest among the four groups at any time point. Meanwhile, the gene expressions of ALP, Runx2, and OCN on HA-2 are lower than those of HA-1 (not significant except for ALP). In addition, the gene expressions on SrHA are higher than those on HA.

## 3. Discussion

In this study, four different nanosized HA and SrHA were prepared. The results showed that the aspect ratio of the HA prepared by Ca(NO_3_)_2_ was lower than that prepared by CaCl_2_. It has been proven that using complexing agents or surfactants can arrest HA crystal growth [30]. Here, it seems that the *a*-plane growth of the HA is inhibited by Cl^−^, whereas the *c*-plane is inhibited by NO_3_^−^. In addition, SrHA nanorods greater than 100 nm in length were prepared by furnace cooling (providing enough time for crystal growth). 

The in vitro experiment showed that the biological performance of SrHA-2 (larger than 100 nm) is slightly better than that of SrHA-1 (but not significantly so). As is well known, SrHA possesses good biocompatibility. However, it would cause cytotoxicity after phagocytosis if the particle size was too small [31]. In the present study, SrHA-2 exhibited the largest size, which might make it unsuitable for filtrating cell membranes, thus, showing a better biological performance than that of SrHA-1. Meanwhile, SrHA-1 exhibited better cell proliferation and differentiation than HA-2. In this study, there were two main factors that might affect the biological performance: particle size and releasing ions. Due to the particle size of HA-2 being similar to that of SrHA-1, the difference could be attributed to the releasing ions of HA-2 and SrHA-1. The SrHA exhibited better cell proliferation and differentiation than HA, which could mainly be attributed to the release of Sr^2+^. Xin et al. found that nanotube arrays incorporated with Sr (without destroying the structure) could improve the biocompatibility and bioactivity [32]. In addition, HA-1 showed better bioactivity than HA-2. This can be explained in two ways. Firstly, the aspect ratio of HA-1 was lower than that of HA-2, which meant that HA-1 is close to spherical in shape. Zhao et al. found that sphere-like nano-HA was beneficial for filopodia protrusion, showing more favorable properties than rod-like HA for osteoblasts [33]. For another, HA-2 showed the fastest releasing rate of all samples, which could be attributed to its high specific surface area [34]. HA dissolving would release calcium ions into the cytoplasm. The proper Ca^2+^ concentrations favored osteoblast proliferation and differentiation [35]. However, increased Ca^2+^ concentrations might trigger apoptosis in osteoblasts [31,36]. Although there is no significant difference between HA-1 and HA-2 at the later stages, the biological differences caused by the nano-scale dimensions should be considered before clinical applications are attempted. 

The current study evaluated the biological performance of nanostructured HA and SrHA with subtle differences in dimensions. The observations showed some evidence for the choice of apatite for orthopedic implant coating. However, there are some limitations and shortcomings that should be addressed in the future. Firstly, some minor soluble phases probably formed, such as brushite (being more soluble than HA), which might affect the Ca^2+^ ions’ release and thus the cell experimental results. Secondly, in this study, dopamine hydrochloride was used (as a binding agent) to fabricate the Ti-based HAP coatings. Hence, the coatings contained Cl and C, as shown in the EDS spectra (Figure 6). The existence of Cl and C might influence the biological performance of the coatings. Thirdly, as is well known, nanosized particles were prone to conglomerate, which might partly interfere with the experimental results. The cytocompatibility was evaluated by an MTT test with MG63, which is an osteosarcoma cell line. The results might be more convincing if some other osteblastic and stem cell lines were chosen and compared. Furthermore, the expressions of some osteogenic genes were determined by RT-PCR, which reflected changes in them at the RNA level. It would be much better if quantitative real-time PCR was applied or Western blot was performed to quantify the related genes at the protein level. Further study is needed to explore the mechanism of nanosized ceramics’ functions on cellular behavior.

## 4. Experimental

### 4.1. Preparation of Samples

**Scheme 1:**
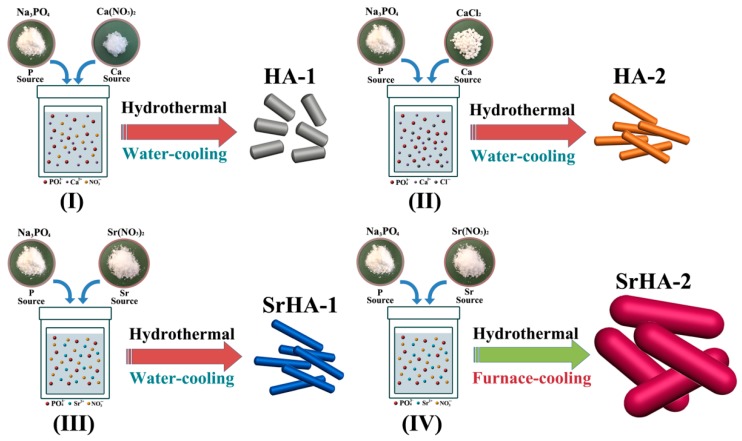
Nanostructured hydroxyapatite with different dimensions via different routes.

All the samples were synthesized by a one-step hydrothermal method, as previously reported [37]. For sample HA-1, Ca(NO_3_)_2_ and Na_3_PO_4_ with a molar ratio of 10:6 were each dissolved in 80 mL of deionized water. Then, the Ca(NO_3_)_2_ solution was added dropwise into the Na_3_PO_4_ solution. After vigorous agitation for 10 min, the suspension was hydrothermally treated at 150 °C for 5 h and then water-cooled. For sample HA-2, the procedure was the same as for sample HA-1, but the Ca(NO_3_)_2_ was replaced by CaCl_2_. For sample SrHA-1, Ca(NO_3_)_2_ was replaced by Sr(NO_3_)_2_. For sample SrHA-2, Ca(NO_3_)_2_ was replaced by Sr(NO_3_)_2_ and water-cooling was replaced by furnace cooling. The precipitates were washed with deionized water and dehydrated absolute ethanol twice. Then, the product was placed in an oven and heated at 80 °C for 24 h for drying. Subsequently, the dried powder was manually ground with a corundum mortar (Scheme 1).

### 4.2. Characterization of the Samples

Phase analysis of the synthesized powders was conducted via X-ray powder diffraction (XRD) and X-ray photoelectron spectroscopy (XPS). The XRD patterns were provided by a Bruker D8 ADVANCE X-ray diffractometer (Karlsruhe, Baden-Württemberg, Germany) equipped with graphite-monochromatized Kα radiation (λ = 1.5418 Å). The diffractometer was operated at 40.0 kV and 30.0 mA with a 2θ range of 10–90° (step size, 0.02) and exposure of 50 s.

The crystallinity degree *X*_c_, corresponding to the fraction of crystalline phase present in the examined volume, was evaluated by Equation (1) [38]:

(1) $$Xc\;\approx \;1\;－\;\left(V_{112/300}/I_{300}\right)$$

where ***I***_300_ is the intensity of (300) reflection and *V*_112/300_ is the intensity of the hollow between (112) and (300) reflections, which completely disappears in non-crystalline samples.

In order to examine the functional groups of the obtained powder, infrared spectra of all samples were obtained using an infrared Fourier-transform spectrometer (FT-IR, Bruker TENSOR 27, Karlsruhe, Germany) in the range of 4000–400 cm^−1^. The powder was ground with KBr to the proportion of 1/150 (by weight) and pressed to a wafer of 13 mm diameter using a hand press.

The powder morphology was observed via scanning and transmission electron microscopy (SEM; TEM). An accelerating voltage of 5 kV was chosen for SEM analysis and the micrographs were captured using secondary electrons collected with an in-lens detector. TEM images were acquired using a JEM-2100F microscope (Akishima, Tokyo, Japan) at 200 kV. High-resolution imaging (HRTEM) and selected-area electron diffraction (SAED) patterns were obtained. 

The specific surface area of powders (m^2^/g) was determined by the Brunauer–Emmett–Teller (BET) method using a Surface Area Analyzer (TriStar 3000, Micromeretics, Norcross, GA, USA) after pre-preparation of samples by heating at 200 °C in a stream of N_2_ in excess of 24 h.

The dimensions of the synthesized particles, including long axis, short axis, and aspect ratio, were obtained by manual measurement of a representative sample (*n* > 500) by TEM.

### 4.3. Cell Experiments

#### 4.3.1. Preparation of HA Coatings on Titanium

Dopamine-assisted immobilization of the synthesized samples onto Ti substrates was carried out, as reported previously [39]. In brief, 3-Hydroxytyramine hydrochloride (dopamine hydrochloride, Sigma Aldrich, Saint Louis, MO, USA) was dissolved in 10 mM Tris buffer (pH 8.5) to 2 mg/mL, while each sample was suspended in the same buffer to 2 mg/mL. Then, the two solutions were mixed (1:1) and 100 µL of the mixture was dropped onto polished Ti plates (10 mm × 10 mm × 1 mm) for 12 h. These modified Ti plates with HA and SrHA coatings were used for cell culture.

#### 4.3.2. Ca^2+^ and Sr^2+^ Release

To examine the release behavior of Ca^2+^ and Sr^2+^ from the coatings, each specimen was immersed in 10 mL of phosphate-buffered saline (PBS) at 37 °C. The solution was refreshed at each time point for seven days. The PBS containing the released Ca^2+^ and Sr^2+^ was analyzed via inductively coupled plasma mass spectroscopy (ICP, VISTA-MPX).

#### 4.3.3. Cell Seeding and Culture Conditions

All the samples (the aforementioned Ti plates) used in the cell culture experiments were first sterilized in an autoclave (at 121 °C for 1 h) and then inserted into 12-well polystyrene cell culture plates (TPP, Trasadingen, Switzerland; internal well diameter: 22.0 mm). Then they were seeded with human osteoblast-like MG 63 cells (European Collection of Cell Cultures, Salisbury, UK) and suspended in Dulbecco’s modified Eagle's minimum essential medium (DMEM; Sigma Aldrich, Cat. N° D5648) with 10% fetal bovine serum (FBS; Sebak GmbH, Aidenbach, Germany) and gentamicin (40 µg/mL, LEK, Ljubljana, Slovenia). Each well contained 36,000 cells (i.e., approximately 10,000 cells/cm^2^) and 2 mL of the medium. The cells were cultured for one, three, or seven days at 37 °C in a humidified air atmosphere containing 5% CO_2_.

#### 4.3.4. Cell Distribution and Proliferation

MTT assay was performed to estimate the cell proliferation. MG-63 cells were seeded in a 12-well plate and incubated for one, three, or seven days. After the selected incubation periods, the samples were washed with phosphate-buffered saline (PBS) and transferred to a new 12-well plate. Then, 300 µL of the culture medium and 300 µL of MTT (3-[4,5-dimethylthiazol-3-yl]-2,5-diphenyl tetrazolium Bromide) reagent (5 mg/mL in PBS, Sigma Aldrich) were added to each well. After 4 h of incubation in a 5% CO_2_ incubator at 37 °C, the medium was replaced with 500 µL of dimethyl sulfoxide to dissolve formazan. The plate was shaken for 10 min, and then the solution in each well was transferred to a 96-well ELISA plate. The optical density (OD) of the dissolved solute was measured using an ELISA reader (Tecan, Salzburg, Austria) at 570 nm (*n* = 9 in each group). The common OD value of the blank group (*n* = 9) was subtracted from the OD value of each group at each time point. The blank group was treated with the same procedures and incubated for the same time as the above groups. 

Representative fluorescence microscopy images of the MG63 cells were obtained to evaluate the cell adhesion and distribution. After seeding for one and three days, the samples were rinsed with PBS (Sigma Aldrich) and stained with Hoechst #33342, which stains the cell nuclei (excitation max., 346 nm; emission max., 460 nm; Sigma Aldrich; 5 µg/mL of PBS). This dye was applied for 2 h at room temperature. The microscopy images were acquired using an IX-51 microscope equipped with a digital camera (DP-70, Olympus, Tokyo, Japan).

#### 4.3.5. Alkaline Phosphatase Activity

MG-63 cells were seeded in a 12-well plate (approximately 10000 cells/cm^2^) with 1 mL of the biomaterial extracts containing 10% fetal bovine serum. After culturing for three, seven, or 14 days, the cultural medium was carefully removed and the plates were gently washed twice with PBS. Then, 500 µL of 0.2% (*v*/*v*) Triton X-100 (Sigma Aldrich) was added to each well. After lysis in a 5% CO_2_ incubator for 2 h, the solutions were transferred to a micro-centrifuge tube and frozen at −80 °C for 2 h. Three freeze–thaw cycles were completed to homogenize the solutions. Then, 3 mL of Coomassie Brilliant Blue staining solution, 0.6 mL of cell lysis solution, and 0.4 mL of double distilled water were mixed and allowed to stand for 10 min. The OD value of the mixed solution was measured using an ELISA reader (Tecan, Salzburg, Austria) at 595 nm. The protein concentration of the cell lysis solution was calculated on the basis of a standard curve obtained using bovine serum albumin as a standard. Then, 100 µL of the cell lysis solution and 100 µL of 25 µg/mL *p*-nitrophenyl phosphate disodium salt (PNPP) were added into each well of a 96-well plate (eight wells in each group). After 30 min, 50 µL of 3 mol/L NaOH was added to terminate the reaction. In the blank group, 100 µL of 0.2% (*v*/*v*) Triton X-100, 100 µL of 25 µg/mL PNPP, and 50 µL of 3 mol/L NaOH were added. The OD values were measured at 405 nm. The OD per milligram of protein was calculated.

#### 4.3.6. Osteogenesis-Related Gene Expression

The expressions of osteogenesis-related genes were evaluated on the basis of a real-time polymerase chain reaction (real-time PCR). The cells were seeded with 2 × 10^4^ cells/well and cultured for three, seven, or 14 days. The total RNA was isolated using the TRIzol reagent (Gibco). Here, 1 mg RNA from each sample was reverse transcribed into complementary DNA (cDNA) using the PrimeScript™ RT reagent kit (TaKaRa). The forward and reverse primers for the selected genes were the same as those described in [40]. The expressions of osteogenesis-related genes, including ALP, runt-related transcription factor 2 (Runx2), and osteocalcin (OCN), were quantified on the basis of real-time PCR (Bio-Rad iQ™5 multicolor real-time PCR detection system, Hercules, CA, USA) with SYBR^®^ Premix Ex™ Taq II (TaKaRa, Dalian, China). Data analysis was carried out using the iQ™5 optical system software version 2.0 (Bio-Rad). The relative expression levels for each gene of interest were normalized to the level of the housekeeping gene GAPDH.

### 4.4. Statistical Analysis

At least three samples were employed for each analysis. All data were statistically analyzed using one-way ANOVA and Tukey’s post hoc comparison to evaluate statistically significant differences between the sample groups. The quantitative data was presented as mean ± standard deviation (SD). The value *p* ≤ 0.05 was considered significant. Dedicated software such as DigitalMicrograph 365 (for PC) (Gatan, Inc., Pleasanton, CA, USA) and MicroCall Origin (Pleasanton, CA, USA) were employed for image processing and mathematical data computation.

## 5. Conclusions

In this study, nanosized HA and SrHA with different dimensions were synthesized and their biological performance on osteoblast-like cells was evaluated. Our results demonstrate that nanosized HA and SrHA with subtle differences could affect the cellular growth. This work provides a view of the role of nano-HAs as ideal biocompatible materials in future clinical applications.

## Figures and Tables

**Table 1:** Crystallinity and specific surface area of the synthesized samples.

Samples	Crystallinity	Specific Surface Area (m^2^/g)
**HA-1**	55.4	57 ± 6
**HA-2**	35.6	92 ± 8
**SrHA-1**	71.9	45 ± 5
**SrHA-2**	83.7	38 ± 4
